# 

**Published:** 1880-10-20

**Authors:** 


### TABLE OF CONTENTS.

ORIGINAL AND SELECTED ARTICLES.

Some Cases Curious of Diagnosis, by A. F. Kinne, A.M., M.D., Mich., 353. A Remarkable Case of Poisoning, by A. F. F. Kerstan, M.D., Atlanta, 358. Bites of Reptiles, by J. Hendree, M.D., Ala., 359. Use of Pessaries, Etc., by I. T. Suggs, M.D., 360. Too Much Materia Medica, Etc., by A. G. Smythe, M.D., Miss., 361. The Physiological Action of Fatty Inunctions, by W. K. Harrison, M.D., 364. On the Influence of Maternal Shock in the Production of Fœtal Monstrosities, by Thos. Wilson, M.R.C.S., L.R.C.P., 365. Water as a Prophylactic and a Remedy, by S. G. Webber, M.D., 368.

ABSTRACTS AND GLEANINGS.

Successful Removal of a Uterine Fibroid with Thomas' Scoop or Spoon, 370. Diseases of the Eye Occurring in Connection with Pregnancy, 371. Abortions, 372. Acidity as a Cause of Sterility; Influence of Tobacco-smoking upon Health, 373. Obstetric Practices Among the Indians, 374. Cupping in Carbuncle; Uterine Dyskinesia and the Treatment of Displacements, 375. Specular Examination of the Rectum; Treatment of Lung Diseases by Condensed Air, 376. Eczematous Crusts; Metaphosphoric Acid a Delicate Test for Albumen in Urine, 377. Administration of Iodide of Potassium; Benzol in Whooping Cough; Prolonged Absence from Food, 378. Systemic Poisoning by External Application of Carbolic Acid; Anthrax of Fruit-trees; Method of Restoring the Asphyxiated, 379. Podophyllin; Action of Quinine, Digitaline and Atropine; No Yellow-fever Germ, 380. Substitute for Tracheotomy; High Temperature from Constipation; For Night-sweats of Patients Suffering with Lung-phthysis; Friction in Insomnia, 381. Neuralgia of the Testis, 382.

SCIENTIFIC ITEMS.

Rather Old Butter; An Electric Alarm for Firemen; Electricity in the Human Body, 383. Animal Electricity; Atoms, 384.

PRACTICAL NOTES AND FORMULÆ.

Treatment of Neurasthenia; Vaselin in Gynæcology; To Keep Flies from Horses, 385. Treatment of Poisoning by Strychnia; Surgical Treatment of Epistaxis, 386.

EDITORIAL AND MISCELLANEOUS.

Editorial Notices, 389. Book Notices, 391. Special Notices, 392.

